# Comprehensive analysis of prognostic value and immune infiltration of Regulator of Chromosome Condensation 2 in lung adenocarcinoma

**DOI:** 10.7150/jca.91367

**Published:** 2024-02-11

**Authors:** Hai Lin, Guofu Lin, Lanlan Lin, Jiansheng Yang, Dongyong Yang, Qinhui Lin, Yuan Xu, Yiming Zeng

**Affiliations:** 1 Department of Respiratory Pulmonary and Critical Care Medicine, The Second Affiliated Hospital of Fujian Medical University, Quanzhou, Fujian province, 362000, China.; 2 Respiratory Medicine Center of Fujian Province, Quanzhou, Fujian province, 362000, China.; 3 The Second Clinical College, Fujian Medical University, Fuzhou, China.; 4 Department of thoracic surgery, The Second Affiliated Hospital of Fujian Medical University, Quanzhou, Fujian province, 362000, China.

**Keywords:** RCC1, RCC2, lung adenocarcinoma, TP53, immune infiltration

## Abstract

**Background:** Lung adenocarcinoma (LUAD) incidence and mortality take the leading place of most malignancies. Previous studies have revealed the regulator of chromosome condensation 1 (RCC1) family members played an essential role during tumorigenesis. However, its biological functions in LUAD still need further investigation.

**Methods:** Several databases were applied to explore potential effects of RCC1 family members on LUAD, such as Oncomine, GEPIA, and cBioPortal. Real-time PCR and immunohistochemistry were used to verify the expression of RCC2 in stage I LUAD. H1975 and A549 were selected to explore the biological function of RCC2 in cellular malignant phenotype.

**Results:** The expressions of RCC1 and RCC2 showed marked differences in malignant tissue compared to lung tissue. The higher the expression levels of RCC1 or RCC2 in LUAD patients, the shorter their overall survival (OS). In normal lung tissues, RCC1 expression was highly enriched in alveolar cells and endothelial cells. Compare with RCC1, RCC2 expression in normal lung tissue was significantly enriched in macrophages, B cells and granulocytes. Additionally, RCC2 expression level was correlated with multiple immune cell infiltration in LUAD. Moreover, the mutation or different sCNA status of RCC2 exerted influence on multiple immune cell infiltration distribution. We found that the upregulation of RCC1 and RCC2 were obviously related to TP53 mutation. GSEA analysis revealed that RCC2 was involved in the process of DNA replication, nucleotide excision repair and cell cycle, which might affect tumor progression through P53 signaling pathway. We further elucidated that downregulation of RCC2 could dramatically repress the migration and invasion of LUAD cells.

**Conclusions:** The study demonstrated that RCC1 and RCC2 expression were markedly increased in early-stage of LUAD. Patients with high expression of RCC1 or RCC2 had a worse prognosis. Based on our analysis, RCC1 and RCC2 might exert influence on LUAD process through DNA replication, nucleotide excision repair and cell cycle, as well as cells migration and invasion. Different from RCC1, RCC2 also involved in immune infiltration. These analyses provided a novel insight into the identification of diagnostic biomarker.

## Introduction

Lung cancer not only has the high cancer-related mobility, but also take the leading place in cancer-caused mortality worldwide 1. Non-small cell lung cancer (NSCLC) and small cell lung cancer (SCLC) are the two main types of lung cancer, of which NSCLC is the vast majority 2. Lung adenocarcinoma (LUAD) is the predominant histopathological type of NSCLC 3,4. With the development of cancer treatment techniques, the mortality of lung cancer has been reduced. The 5-year survival rate for lung cancer patients with no metastasis is only 19%, which is nearly double of the group with metastasis 5. It is now widely suggested that molecular alterations are the trigger for these carcinogenic steps, and exposure to environmental pathogenic factors can accelerate cancer growth. 6-9. Therefore, it is of importance to reveal the molecular biological mechanism and identify potential biomarkers for early diagnosis in LUAD.

Members of the Regulator of Chromosome Condensation 1 (RCC1) superfamily are characterized by a RCC1-like domain 10,11. According to the structural characteristics, the members of this superfamily can be divided into five subgroups 12. RCC1, the representative protein of RCC1 superfamily, is a nuclear Ras-like G protein. It can mediate the condensation of chromatin during the cell cycle, especially in the S and M phases. Accumulated evidence demonstrates that mutations of RCC1 were involved in tumor development, and upregulation of RCC1 played an essential role in tumor progression, such as breast cancer and ovarian cancer 13-17.

RCC2, a constituting component of passenger proteins, is essential to facilitate the proper chromosome segregation and cell division 18,19. Like RCC1, RCC2 activity is essential to cell cycle regulation especially in interphase and mitosis 20. Nevertheless, RCC2 is specifically expressed in G2 later phase until mitosis. Different from RCC1, RCC2 only participated in the modulation of tumorigenesis and imposed influences on therapeutic resistance 21. Although RCC2 has been verified to be a susceptibility gene for breast cancer and colorectal cancer, the function of RCC2 in lung cancer is still unclear. In addition to RCC1 and RCC2, deafness locus-associated putative guanine nucleotide exchange factor (DelGEF) and William-Beuren syndrome critical region 16 (WBSCR16) are members of RCC1 superfamily, participating in guanine nucleotide exchange and mitochondrial fusion 22-23. However, studies on the function of DelGEF and WBSCR16 in cancers are still limited.

In this study, we conducted research on RCC1 subgroup genes using multiple online databases to detect the biological and prognostic value in LUAD. We found that RCC1 and RCC2 expression levels are significantly increased in LUAD, while DelGEF and WBSCR16 expressions did not change. Up-regulation of RCC1 and RCC2 was associate with worse prognosis. RCC2 exerted an influence on the immune cell infiltration in LUAD while RCC1 did not. We also explored the molecular mechanism by which RCC2 mediate the LUAD progression. Meanwhile, we demonstrated that down-regulation of RCC2 can suppress the migration and division abilities of LUAD cells.

## Methods

### Oncomine

Oncomine (www.oncomine.org) is a web-based database and data-mining platform aimed at facilitating new discoveries from genome-wide expression analyses 24. It carries on genes expression comparative analysis between major cancer and their normal tissues. It also contains exploration about the cancer base of clinical and pathology information. The database was selected to analyze RCC1 superfamily members mRNA different expression in LUAD. We set the restrictive thresholds to reduce the mistaken rate including two-fold change of mRNA expression between LUAD and adjacent tissues, with P-value <0.05 and top 10% gene ranked.

### UALCAN Data Analysis

UALCAN (http://ualcan.path.uab.edu) is a comprehensive web tool to facilitate analyze based on MET500 cohort and the Cancer Genome Atlas (TCGA) datasets 25. UALCAN was used to compare the transcriptional level of the RCC1 superfamily members in LUAD tissues and adjacent tissues, then calculating the significant difference using students' t-test, with P <0.05 considered as statistically significant.

### Human Protein Atlas (HPA) Data analysis

The Human Protein Atlas (https://www.proteinatlas.org) is a website which contains twenty kinds of the most common types of cancers with their immunohistochemistry-based expression profiles 26. According to the fraction of stained cells, staining quantity was also divided into four levels. Based on staining intensity and staining quantity, the protein expression levels were classified into seven levels. The different protein level of RCC1 and RCC2 in LUAD and adjacent normal tissues were assessed by HPA. Single cell RNA analysis was used to evaluate the distribution of RCC1/RCC2 in normal lung tissue and human immune cells.

### GEPIA Data Analysis

GEPIA (http://gepia.cancer-pku.cn/) is an interactive web that covers 8,587 normal and 9,736 cancer tissues arising from TCGA and Genotype-Tissue Expression (GTEx) projects 27. We selected the TCGA database to evaluate the expression of RCC1 and RCC2. According to the log-rank and Mantel-Cox tests result, we separate the patient into high- and low-expression subgroup. 458 patients were chosen to assessed the overall survival (OS) and disease-free survival (DFS). Then, we examined the Hazard ratio (HR), 95% confident interval (CI), and P value according to the result above.

### Kaplan-Meier Plotter

Kaplan-Meier plotter (http://kmplot.com/analysis) is introduced to determine the prognostic value of the mRNA expression of RCC1 and RCC2 in LUAD patients 28. We carried on the analysis about overall survival (OS), first-progression survival (FPS), and post-progression survival (PPS) between high- and low-expression group of LUAD patient.

### Univariate and Multivariate Cox Regression

RNA-sequencing expression (level 3) profiles and corresponding clinical information for LUAD were downloaded from the TCGA dataset (https://portal.gdc.com). Univariate and multivariate cox regression analysis were performed to identify the proper terms to build the nomogram. The forest was used to show the P value, HR and 95% CI of each variable through 'forestplot' R package.

### GeneMANIA

GeneMANIA (http://www.genemania.org) is an online predicting tool to explore the genetic and protein interactions, co-expression, co-localization, and domain-protein similarity of target genes 29. Using this database, we conducted the network between RCC1/2 and their interactive genes.

### cBioPortal Data Analysis

cBioPortal (www.cbioportal.org) is a comprehensive web resource, with visualizing and analyzing multidimensional cancer genomic datasets based on TCGA database 30. We obtained the genetic alterations and the network modules of RCC1 and RCC2 from cBioPortal. In the present study, the TCGA lung adenocarcinoma (TCGA, Firehose Leagacy) dataset, including 230 samples with complete information about RCC1 and RCC2 expression, was selected for analysis.

### Metascape Analysis

Metascape is a comprehensive online tool for genetic annotation and enrichment analysis 31. Through gene ontology (GO) and Kyoto Encyclopedia of Genes and Genomes (KEGG) 32, the functions of the RCC1 and RCC2 and their co-expression genes were enriched into different biological activities. The threshold value was set as 0.01, and the enrichment factor of > 1.5 and a minimum count of 3 were considered as significant. Meanwhile, we predicted the transcriptional factor which interact with the above genes.

### STRING

STRING (https://string-db.org/) is a website about protein interaction, whose ability is to collect, score, and integrate all publicly available sources of protein-protein interaction (PPI) data 33. Through collection and interaction of the different expression genes, we built a PPI network which center is RCC1/2 and predicted their potential interaction relationship.

### TIMER

TIMER (https://cistrome.shinyapps.io/timer/) is an intuitive website which is applied to systematically evaluate the correlation between the relative gene expression with infiltration of various immune cells and their clinical effects 30. We evaluated association between RCC2 expression level and infiltrating immune cells in LUAD. The effect of RCC2 mutation and different sCNA status of RCC2 on immune cell infiltration were assessed by this database.

### GSEA

Gene set enrichment analysis (GSEA) performs its functions by focusing on gene sets which are groups of genes sharing common biological function, chromosomal location, and regulation. Through GSEA, we assessed the influence of RCC2 on DNA replication, nucleotide excision repair, cell cycle, and association with p53 signaling pathway in LUAD.

### Gene expression omnibus (GEO) expression dataset analysis

In the GEO database (https://www.ncbi.nlm.nih.gov/geo/), expression data sets related to LUAD were obtained, namely GSE31548 (30 LUAD cases and 20 normal cases). Gene expression was calculated using | log2FC | >1 and FDR <0.05 as criteria for significant difference using the R statistics package 'limma'. The box plot of RCC2 expression is implemented by the R software package 'ggplot2'.

### PCR

The total RNA of 32 paired stage I LUAD and adjacent normal tissues was extracted using TRIzol as the established protocol. The Takara PrimeScript™ RT reagent kit (Takara, Japan) was used to synthetize cDNA from 1,000 ng RNA. TB Green Mixture (Takara Bio, Japan) was used to conduct RT-PCR using QuantStudio™ 5 Real-Time PCR Systems (Applied Biosystems). Every sample was assessed in triplicate. GAPDH was used to normalize the amount of cDNA between different samples. Primer sequences were listed as follows: RCC2 forward 5'-GGAGCTGGGTTTAGAACG-3'; reverse 5'-TTCTAGTGCTTCCAAGCTCCTA-3'; GAPDH forward 5'-ATCACTGCCACCCAGAAGAC-3'; reverse 5'-TTTCTAGACGGCAGGTCAGG-3'.

### Western Blot

Western blot was performed according to the standard protocol. Proteins were extracted from cells and tissues with RIPA buffer (Beyotime, P0013B) complemented with protease inhibitors (Beyotime, P1005). Protein extracts were subjected to electrophoresis on 5× SDS-PAGE and transferred onto PVDF membranes (Immobilon: Millipore, IPVH00010). Immunoblots were blocked with 5% milk solution in TBS-Tween containing 0.1% Tween 20 and incubated with Anti-RCC2 antibodies (Abcamab154705 1:2000) or Anti-GADPH antibodies (Abcam ab181602 1:5000) overnight at 4℃. Subsequently, an appropriate HRP-conjugated secondary antibody (Beyotime A0216 1:5000) was added followed by incubation at room temperature for 1h. Western blots were visualized with chemiluminescence reagents (Sigma, RPN2106).

### Immunohistochemistry

The tumor tissue and their adjacent normal tissue from 58 stage I LUAD patients were selected for immunohistochemistry assay. Paraffin-embedded sections were dewaxed and rehydrated in a series of alcohol to PBS. Then the slides were soaked in 0.1 mol/L citrate buffer (pH 6.0) and placed in an autoclave at 121℃ for 3 minutes for antigen retrieval. After washing with PBS (pH 7.4) for 3 times, tumor sections were blocked with 1% BSA diluted in PBS at 37°C for 30 minutes and incubated with Anti-RCC2 antibodies (Abcam ab154705 1:200) overnight at 4°C. Then the HRP-conjugated goat anti-mouse/rabbit antibody and DAB (DAKO, Glostrup, Denmark) were used. Finally, the sections were counterstained by hematoxylin and mounted.

### Cellular Culture

The human lung adenocarcinoma cell line A549, H1975, H1299, and H460 was cultured in RPMI 1640 supplemented with 10% FBS and 100 U/mL penicillin/streptomycin. The cells above were bought from ATCC (http://www.atcc.org/en/Support/Find_ATCC_Distributors.aspx). The human lung adenocarcinoma cell line SPCA-1 and the human normal airway epithelium cell line BASE-2B was cultured in RPMI DMEM supplemented with 10% FBS and 100 U/mL penicillin/streptomycin. All the cellular assays were performed at 37 °C, 5% CO^2^ and humidity conditions.

### Construction of Stable Transfer Cell Line

Search the mRNA sequence of the target gene by NCBI, design primers and enzyme cleavage sites for the CDS region and the selected vector. The sh-RNA fragment was synthesized and purified by Shanghai Ji Kai Gene Technology Co., Ltd. Cultured lung adenocarcinoma cells A549 and H1975 in 6-cm culture dishes, inoculating cells at a density of 70%-80% cell fusion on the second day, with a volume of 2 mL of culture medium, and incubate overnight at 37°C in a 5% CO^2^ incubator. When the cells grow to logarithmic growth phase, the virus was added to the lung adenocarcinoma cells to be infected by diluting the medium without FBS and adding 8 μL of polybrene at a concentration of 1 mg/mL to enhance the infection effect. After 24 h of virus infection, the medium was changed to RPMI1640 medium containing 10% FBS with optimal concentration of puromycin. Another control dish with medium containing puromycin without viral solution was set up as a control for the effectiveness of puromycin. Thereafter, we changed the medium every other day using a complete medium containing puromycin to replace the medium containing a large number of dead cells until the resistant community could be identified. After the cells in the 6-cm dish were full grown, the cells were harvested and the mRNA and protein levels of RCC2 were detected using RT-qPCR and Western blot, respectively, to assess the infection efficiency.

### Wound-Healing Assay

To examine the effects of RCC2 on the migration of LUAD cells, A549 and H1975 cells were planted in 6 -well culture plates (1.0×10^6^ cells). Both cells were culture until 80-90% confluency was achieved. A pipette tip was used to scratch a wound in a well, and washing was performed to remove unattached cells. After cell were treated with RPMI 1640 without FBS, the wounds were photograph at 0 and 48 h. Then compared the migration distance between sh-NC cell and sh-RCC2 cell.

### Transwell Assay

The effect of RCC2 on the migration and invasion abilities of LUAD cells was detected by transwell assay. Sh-RCC2 cells and sh-NC cells were conventionally digested and suspended in RPMI 1640 medium without FBS. After counting the cell density, they were seeded into the upper chamber and RPMI 1640 supplemented with 20% FBS were added to the lower chamber. For invasion assay, the upper chambers were coated with diluted Matrigel (BD Biosciences). After incubation for 24 h, the cells were fixed with paraformaldehyde (4%), stained with crystal violet (0.5%), and photographed under a microscope.

### Statistical Analysis

The data shown as the mean ± standard deviation (SD) were obtained by at least three independent experiments and analyzed by the GraphPad Prism 8.0.0 and SPSS 22.0 software. The statistical significance of observed differences was analyzed by ANOVA test. All statistical significance was considered at P < 0.05.

## Results

### Differential Expression of RCC1 Superfamily Genes in LUAD

**Figure 1:**
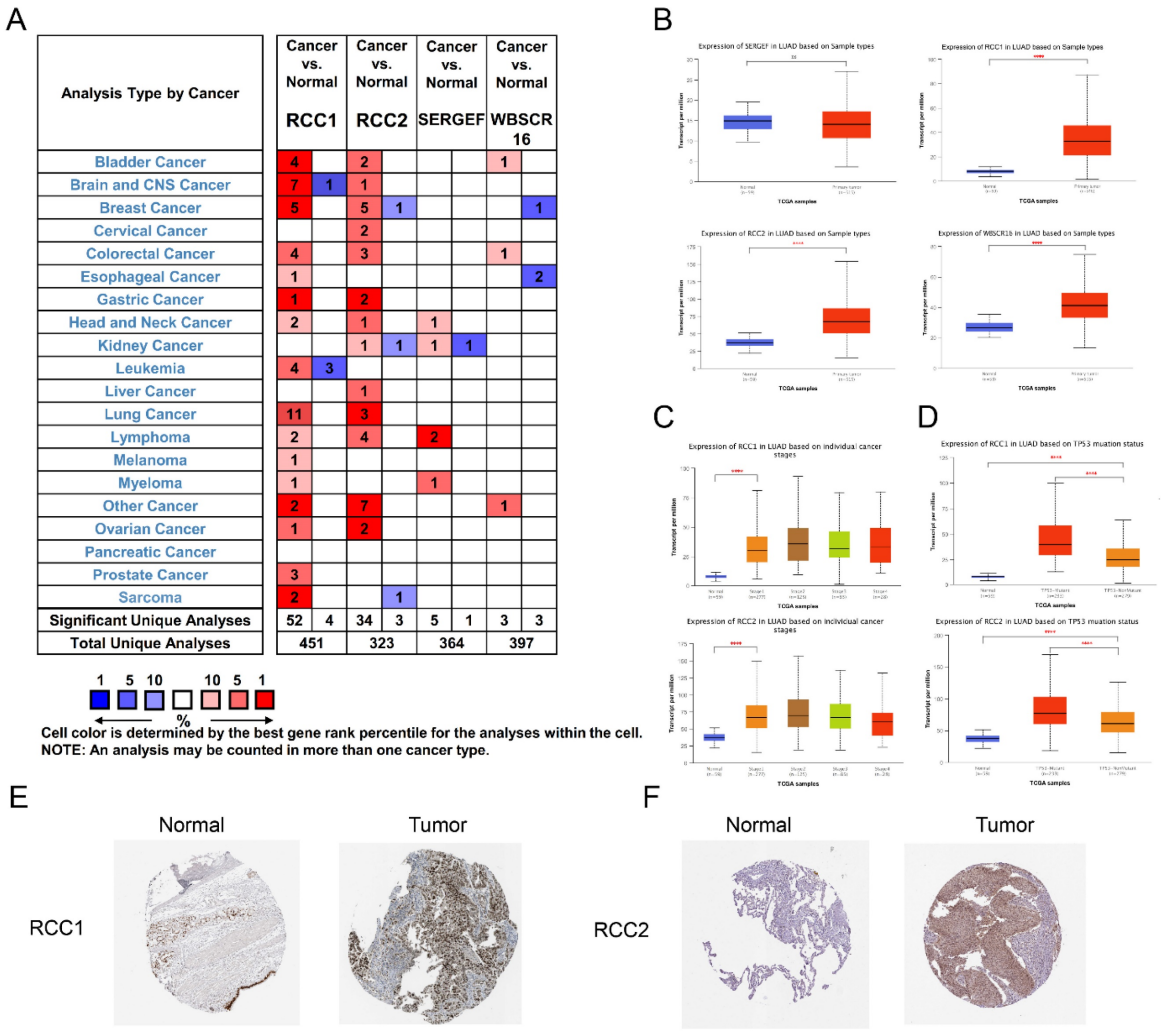
The mRNA level of distinct RCC1 superfamily members in LUAD tissues and normal lung tissues. (A) The transcription levels of RCC1 superfamily in different cancer types (Oncomine): upregulated (red) and downregulated (blue). The following criteria were used: p value < 0.05; fold change absolute value < 2, gene rank: 10%. (B) Expression of RCC1 superfamily members in LUAD tissues and normal lung tissues (UALCAN). (C) Correlations between RCC1 and RCC2 expression and tumor stage of LUAD patients (UALCAN). (D) Relationship between RCC1 or RCC2 mRNA level and LUAD patient with TP53 mutation (UALCAN). (E) The protein expression level of RCC1 in LUAD tissues and normal lung tissues (HPA). (F) The protein expression level of RCC2 in LUAD tissues and normal lung tissues (HPA). (ns means p > 0.05; **** means p < 0.0001)

The mRNA expression levels of RCC1 superfamily genes in LUAD patients were analyzed using the Oncomine database **(Figure 1A)**. Subsequently, we noticed that the mRNA level of RCC1 was significantly increased in LUAD compared with normal lung tissues, and the expression of RCC2 showed a moderate increase in LUAD, but the WBSCR16 and SERGEF expression level change is not significantly.

Also, the expression level of RCC1 superfamily genes in LUAD were analyzed by UALCAN. The results indicated that expression of RCC1, RCC2 and WBSCR16 were markedly upregulated in LUAD tissues (P < 0.0001), while SERGEF showed no change** (Figure 1B)**. Taken together, RCC1 and RCC2 were selected for further study. We assessed the expression of RCC1 and RCC2 in different pathological stage of LUAD. RCC1 and RCC2 expression were dramatically elevated in stage I (P < 0.0001) and their expression remained at a stable level in tissues of advanced stage** (Figure 1C)**. In addition, we explored the relevance between TP53 mutation status and the mRNA level of RCC1 and RCC2. As shown in **Figure 1D**, RCC1 and RCC2 expression level were significantly higher (P < 0.0001) in patients with TP53 mutant than the non-mutant patients.

Furthermore, the HPA database was selected to assess the expression change of these genes in LUAD patients from protein level. Obviously, RCC1 and RCC2 expressed higher protein level in tumor tissues than in normal tissues. As shown in **Figure 1E-F**, RCC1 was dramatically upregulated in LUAD, while the level of RCC2 elevated moderately in LUAD.

Taken together, these analyses indicated that RCC1 and RCC2 were overexpressed in LUAD at both transcriptional and protein level. What's more, the mRNA level of RCC1 and RCC2 was elevated at the beginning of the early stage of LUAD, suggesting that they have the potential to be biomarkers which could be applied to the early diagnosis of LUAD.

### The Prognostic Value of RCC1 and RCC2 in LUAD

**Figure 2:**
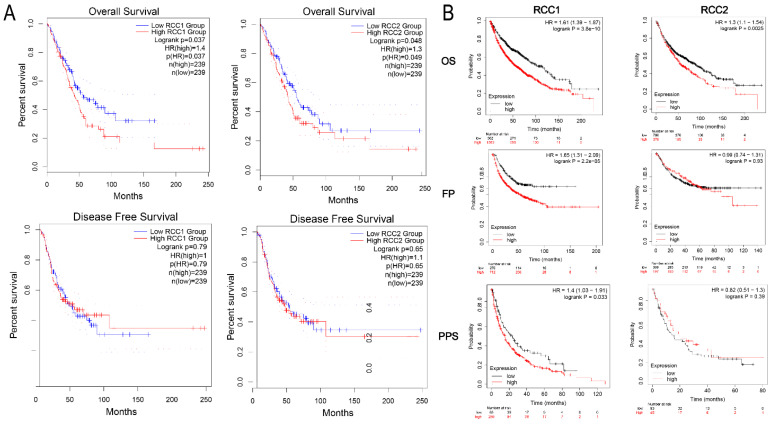
The survival analysis of LUAD patients with different expression level of RCC1 and RCC2. (A) Higher expression level of RCC1or RCC2 was significantly associated with shorter overall survival (OS) in LUAD patients (GEPIA). (B) Different expression of RCC1 or RCC2 exerted impact on LUAD patients' overall survival (OS), first-progression survival (PF) and post-progression survival (PPS) (Kaplan-Meier Plotter).

To evaluate the influence of RCC1 and RCC2 on the LUAD progression, we analyzed the impaction of different RCC1 and RCC2 expression on LUAD clinical outcome by GEPIA. DFS and OS curves were showed in **Figure 2A**. The result suggested that the LUAD patients with overexpression of RCC1 or RCC2 (Logrank P = 0.037, Logrank P = 0.048) would have shorter OS than the patients with lower expression. While, the transcriptional levels of RCC1 and RCC2 did not have impact on DFS of patients.

Additionally, Kaplan-Meier plotter was used to show the association between RCC1 and RCC2 expression level and carcinogenesis, progression, and prognosis of LUAD patients. Consistent with the result obtained from GEPAI, overexpression of RCC1 or RCC2 (Logrank P = 3.8e-10, Logrank P = 0.0025) could dramatically shortened the OS of LUAD patients. In addition, we assessed the effect of the upregulation of RCC1 and RCC2 on the FPS and PPS of LUAD patients. The curves were presented in **Figure 2B**. Higher RCC1 mRNA expression was correlated with shorter PFS (HR = 1.65, P = 2.2e-05) and PPS (HR = 1.4, P = 0.033) in LUAD patients; while the expression of RCC2 did not have such influence on PFS and PPS.

**Figure 3:**
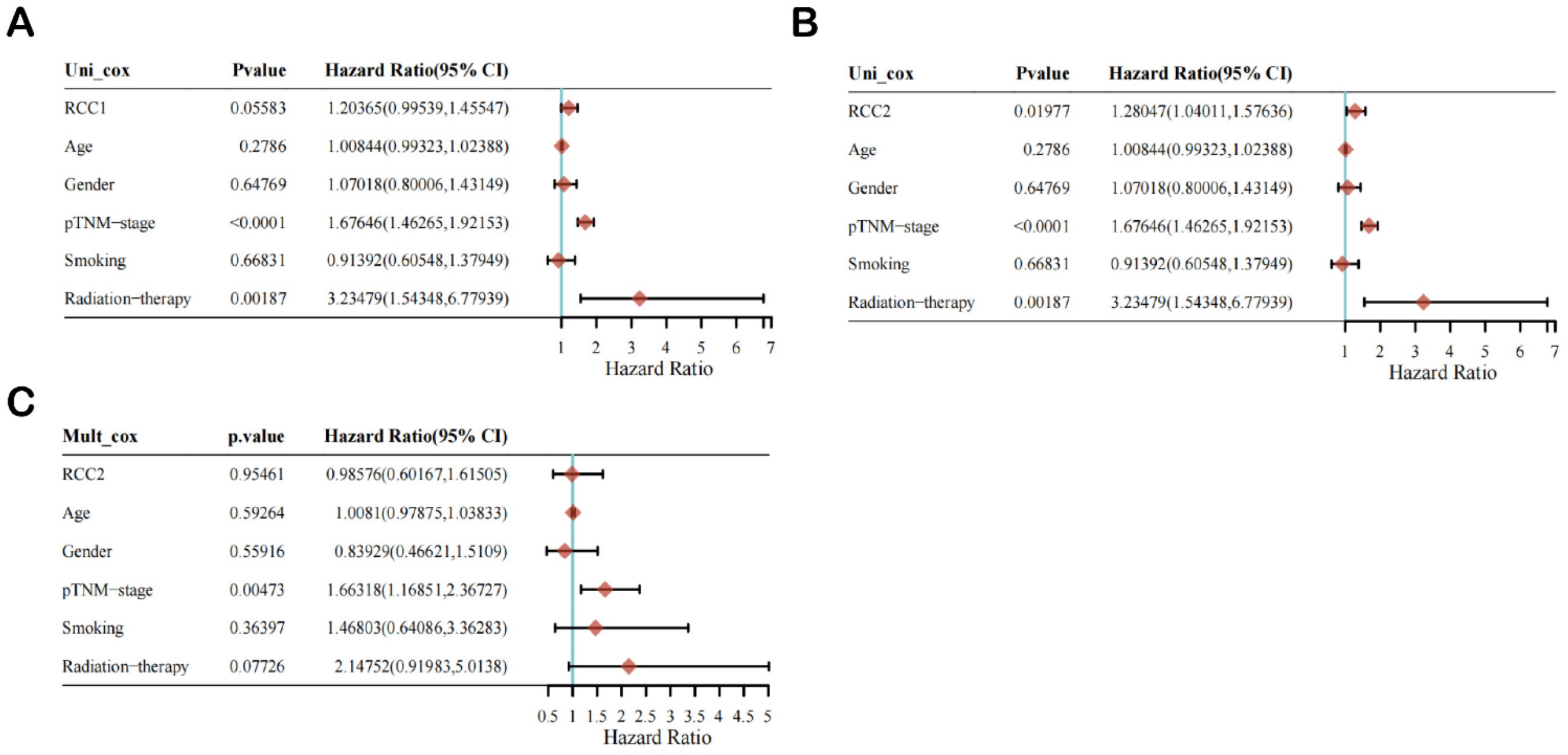
The univariate and multivariate Cox regression analysis of RCC1 and RCC2 in LUAD patients. (A) Univariate Cox regression analysis of RCC1 in LUAD patients. (B) Univariate Cox regression analysis of RCC2 in LUAD patients. (C) Multivariate Cox regression analysis of RCC2 in LUAD patients.

Then, we selected age, gender, pTNM-stage, smoking, and radiotherapy as reference factors to conduct univariate and multivariate Cox regression analysis of the prognostic impact of RCC1 and RCC2 on LUAD patients **(Figure 3)**. Univariate Cox regression analysis result indicated that the expression level of RCC2 (HR = 1.28047 (1.04011, 1.57636), P value = 0.01977) and pTNM-stage (HR = 1.67646 (1.46265-1.92153), P value < 0.0001) is significantly correlated with poor prognosis in LUAD patients **(Figure 3B)**, while RCC1 expression level is not a prognostic factor in LUAD patients **(Figure 3A)**. After that, we selected RCC2 to conduct multivariate Cox regression analysis **(Figure 3C)**. Unfortunately, only pTNM-stage shown a significant difference (HR = 1.66318 (1.16851, 2.36727), P value = 0.00473), which indicated that RCC2 was not an independent prognostic factor in LUAD patients.

### Alterations of the Frequency of RCC1 and RCC2

**Figure 4:**
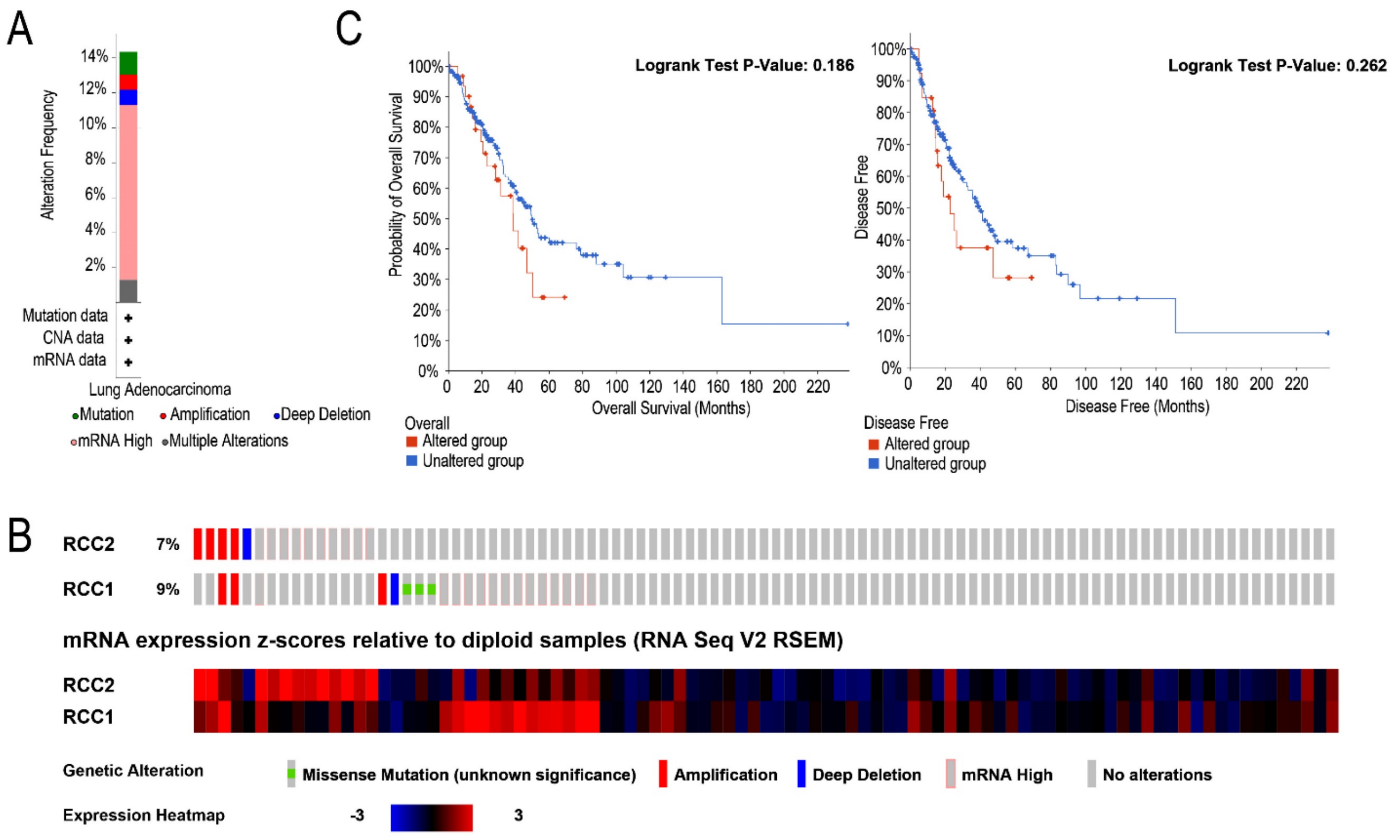
The alteration frequency and prognostic analysis of RCC1 and RCC2 in LUAD were derived from cBioPortal. (A) Summary of RCC1 and RCC2 alteration in LUAD patients. (B) Using the cBioPortal database, we assessed the RCC1 and RCC2 genetic alteration frequency of LUAD samples in TCGA database. (C) The overall survival (OS) between LUAD patients with/without RCC1 or RCC2 alteration was assessed by Kaplan-Meier.

We analyzed the RCC1 and RCC2 alterations by using the cBioPortal online tool for LUAD patients. Totally, RCC1 and RCC2 were altered in 33 samples of 230 LUAD patients (14%) and the concurrent alterations were discovered in only 3 patients (1.4%). Alterations of RCC1 and RCC2 in LUAD patients included amplification, deep deletion, mRNA high, multiple alteration and mutation. As shown in **Figure 4A** mRNA high is the most common genetic change, nearly 10%. Specifically, the percentage of changes in genetic alterations of RCC1 and RCC2 among LUAD patients were 7% and 9%, respectively **(Figure 4B)**. Furthermore, cBioportal dataset were used to assess the associations between RCC1 and RCC2 expression level changes and patients' prognosis** (Figure 4C)**. Unfortunately, according to the Kaplan-Meier curves, neither OS nor DFS exhibited significant correlation with RCC1 and RCC2 mRNA expression alteration.

### Different Expression of RCC2 Altered the Immune Cell Infiltration in LUAD

**Figure 5:**
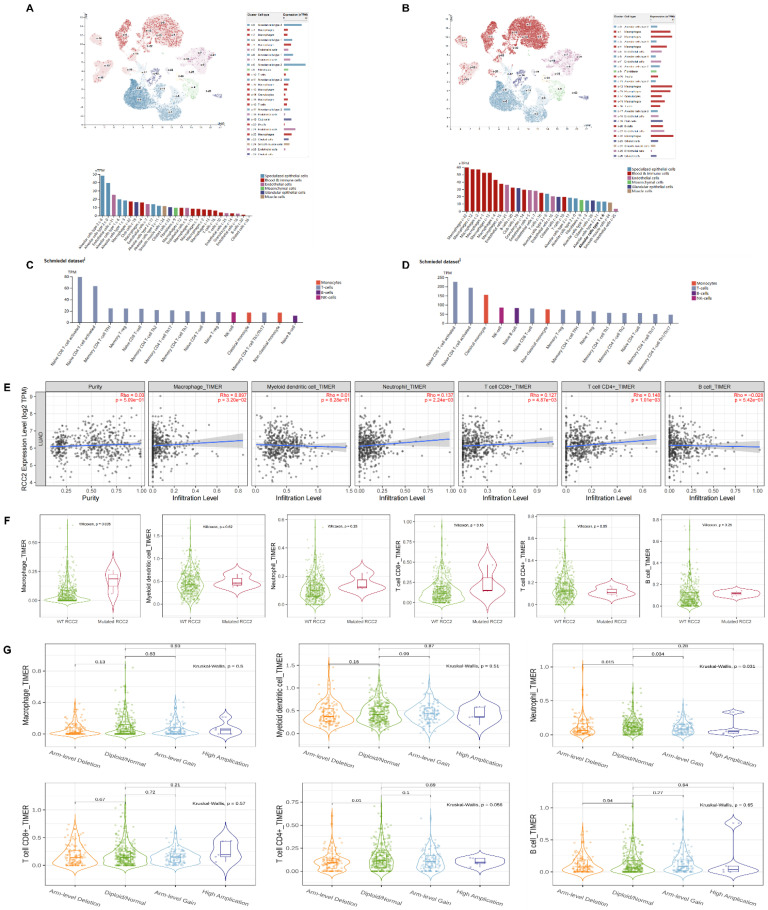
Correlation between RCC1 and RCC2 expression and immune cell infiltration. (A-B) The distribution of RCC1 and RCC2 expression in normal lung tissues was assessed by the single cell RNA analysis in the HPA database. (C-D) The RNA profiles of RCC1 and RCC2 in human immune cells was evaluated by HPA database. (E) Correlation between multiple types of immune cell infiltration and RCC2 expression were detected by TIMER database. The result was adjusted by purity. (F) The TIMER database was used to assess the effect of RCC2 mutation on immune cell infiltration. (G) Comparing immune infiltration distribution based on the sCNA status of RCC2 across TCGA cancer types (TIMER).

Firstly, we carried on single cell RNA analysis to explore the distribution of RCC1 and RCC2 in lung tissue through HPA database. As shown in **Figure 5A**, RCC1 was highly expressed in alveolar cells and endothelial cells. Moderate expression of RCC1 was detected in macrophages. RCC2 expression level was opposite to RCC1. It was significantly enriched in macrophages, B cells and granulocytes **(Figure 5B)**. We then further evaluated the RNA profiles of RCC1 and RCC2 in the immune cells. RCC2 expression level outclassed RCC1 among multiple immune cells, especially in naive CD8 T-cell activated, naive CD4 T-cell activated and classical monocyte **(Figure 5C-D)**.

According to the above results, we selected RCC2 for the further research. The TIMER database was used to investigate the mutual effect between RCC2 expression and immune cell infiltration in LUAD. As shown in **Figure 5E**, RCC2 expression level showed positive correlation with macrophage infiltration, neutrophil infiltration, T cell CD8+ infiltration and T cell CD4+ infiltration (P <0.05). But the relevance was not strong. Then we assessed the effect of RCC2 mutation on immune cell infiltration. Different from previous studies, mutated RCC2 only enhance macrophage infiltration level (P<0.05) but had not influence on other immune cells infiltration level **(Figure 5F)**. Finally, we compared immune infiltration distribution by the sCNA status of RCC2 across TCGA cancer types. Arm-level deletion would lead to neutrophil and T cell CD4+ infiltration decrease (P<0.05). What's more, neutrophil infiltration level can be inhibited by arm-level gain (P<0.05)** (Figure 5G)**. Taken together, RCC2 exerts moderate impact on immune cell infiltration in LUAD.

### Co-expression and Functional Enrichment Analysis of RCC1 and RCC2

**Figure 6:**
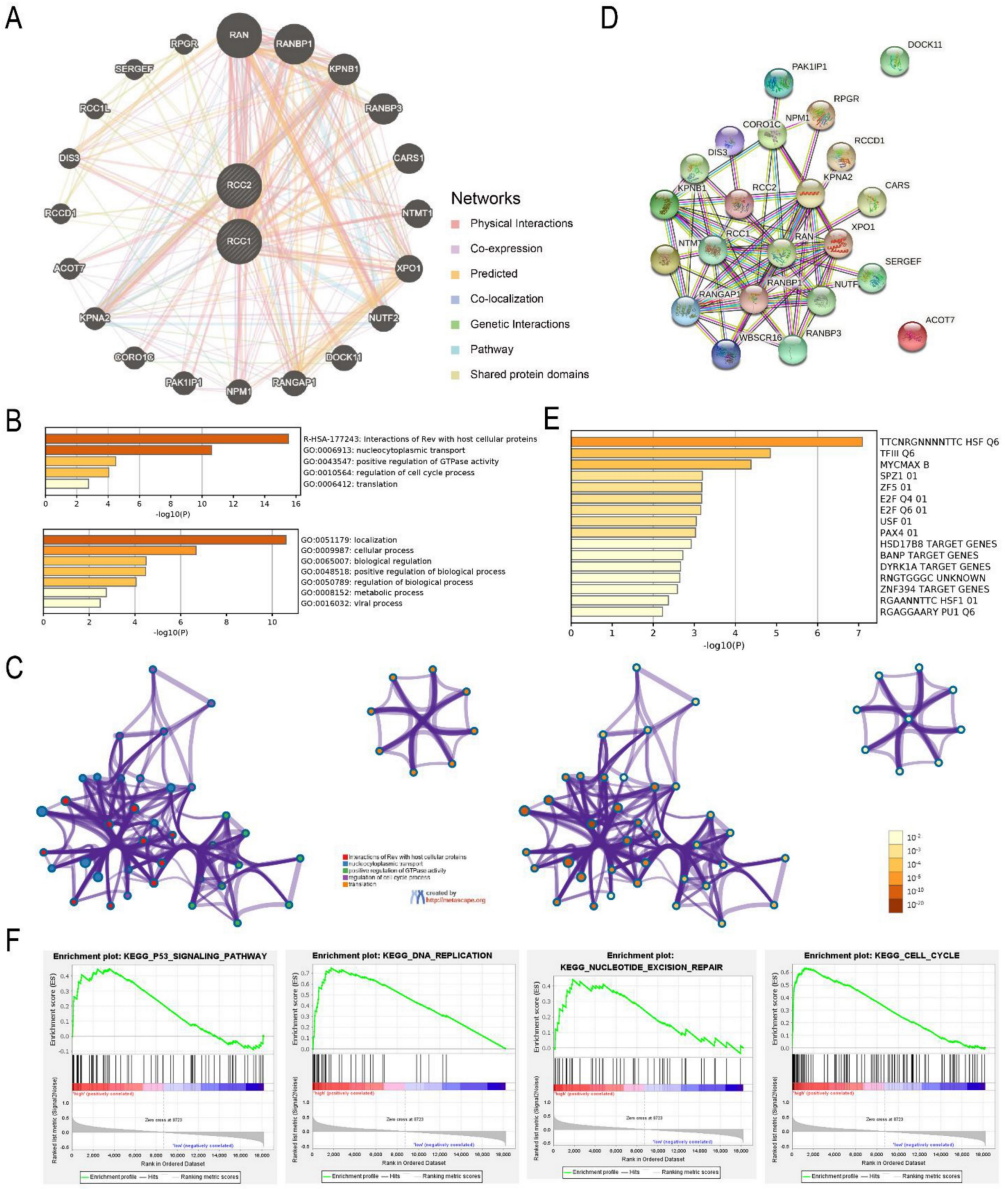
Interaction network and enrichment analysis of RCC1 and RCC2 in LUAD patients. (A) Co-expression network of RCC1 and RCC2 in LUAD (GeneMANIA). (B) The top five functional categories were evaluated by Metascape analysis. (C) Co-relationship among the top five enrich terms, color by cluster ID (Metascape). (D) Protein-protein interaction network of RCC1 and RCC2 and their co-expression genes (STRING). (E) The prediction of transcription factor targets for RCC1, RCC2 and their related genes (Metascape). (F) The mechanism of RCC2 influencing LUAD progression was assessed by GSEA.

To elucidate the molecular mechanism of RCC1 and RCC2 in LUAD, RCC1 and RCC2 and their functional related genes were selected to build an interactional network by GenMANIA. Finally, we selected 20 correlation genes **(Figure 6A)**. Through Metascape database, we predicted the biological functions of RCC1, RCC2 and their co-expression genes. As shown in **Figure 6B**, interaction of Rev with host cellular proteins was the most significant enrichment followed by nucleocytoplasmic transport. In addition to the enrichment analysis, we also built the structure network of enriched terms **(Figure 6C)**. With the help of the STRING online tool, the PPI networks of these genes were constructed to expound the correlation between RCC1 and RCC2 different expression and LUAD **(Figure 6D)**. The result indicated these differentially expressed genes were associated with ribosomal small subunit export from nucleus process and the function of Ran guanyl-nucleotide exchange factor activity. The predicted transcription factor targets were enriched using Metascape **(Figure 6E)**. TTCNRGNNNNTTC HSF Q6 was the most relevant transcription factors, followed by TFIII Q6 and MYCMAX B. The function and mechanism of RCC1 in LUAD had been already tudied, so we carried out the GESA analysis to reveal the mechanism of RCC2 in LUAD. The result indicated that RCC2 might influence the p53 signaling pathway, cell cycle, DNA replication, and nucleotide excision repair** (Figure 6F)**. These results provide a new direction for cancer treatment and drug development.

### Identification the Alterations of RCC2 on LUAD

**Figure 7:**
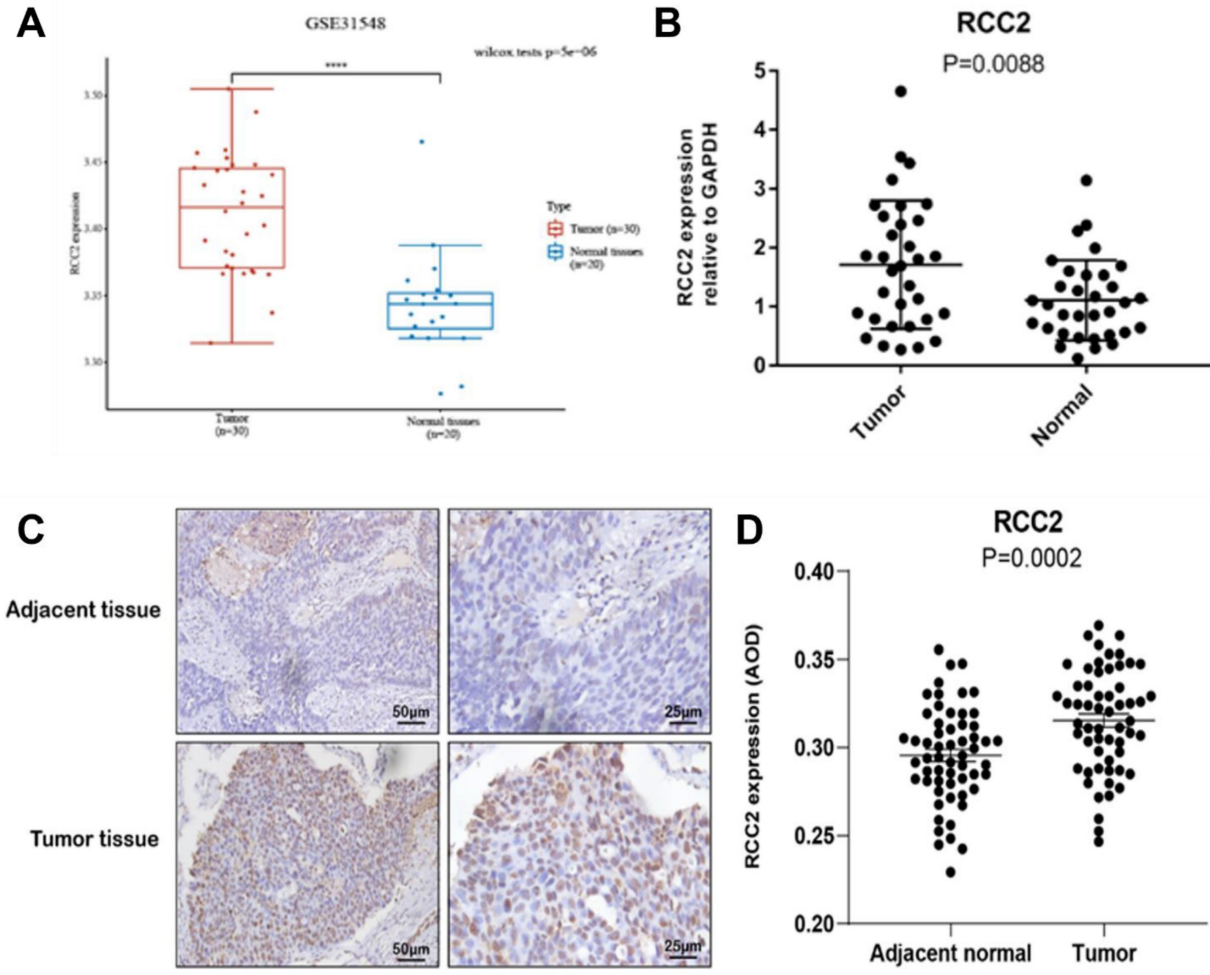
Verification of RCC2 expression in stage Ⅰ of LUAD patients. (A) Comparison of RCC2 expression between LUAD tissues and normal lung tissues by GEO database (GSE31548). (B) Verifying the RCC2 differential expression in LUAD patients by RT-PCR (32 LUAD patients). (C-D) RCC2 shows significantly high expression level in LUAD tumor tissue compared to adjacent tissues, as determined by immunohistochemical assay (58 patients, p < 0.05). (**** means p < 0.0001)

RCC1 can regulate the development of variety cancer, such as breast cancer, ovarian cancer and so on, so we selected RCC2 as research object for the next stage. Firstly, the GEO database was used to certify the RCC2 expression level** (Figure 7A)**. The results demonstrated that RCC2 expression was markedly up-regulated in LUAD as compared with normal lung tissues from two expression datasets (GSE31548). After that, we continued to carry out RT-PCR using tissue from the early-stage LUAD patients** (Figure 7B)**. As expected, the mRNA level of RCC2 was dramatically increased in malignant tumor tissue compared with para-carcinoma tissue (P <0.01). These results clarified that RCC2 was overexpressed in LUAD. Meanwhile, immunohistochemistry was used to verify the protein level of RCC2 in early stage of LUAD** (Figure 7C-D)**. Consistent with the results, protein level of RCC2 showed obvious overexpression in LUAD patients. Taken together, our results indicated that RCC2 could be regarded as a predictive factor of early diagnosis in LUAD patients.

### RCC2 differntial expression mediates LUAD cell malignant phenotype

In order to explore the influence of differential expression of RCC2 on LUAD cell malignant phenotype, we detected the expression level of RCC2 between normal airway epithelial cell line (BASE-2B) and different LUAD cell lines (H1299, H460, SPCA-1, A549 and H1975) to select the suitable cell lines for intervention **(Figure S1A)**. The result indicated that RCC2 expression level increased to a high level in A549 (P<0.0001) and H1975 cells (P<0.05) when compared to BASE-2B cells, thus, A549 and H1975 cell lines was selected for the subsequent experiment. We knocked down RCC2 expression in A549 and H1975 cells **(Figure S1B-S1C)**, then detected the cell migration and invasion ability change aim to clarify the function of RCC2 in LUAD cell malignant phenotype.

**Figure 8:**
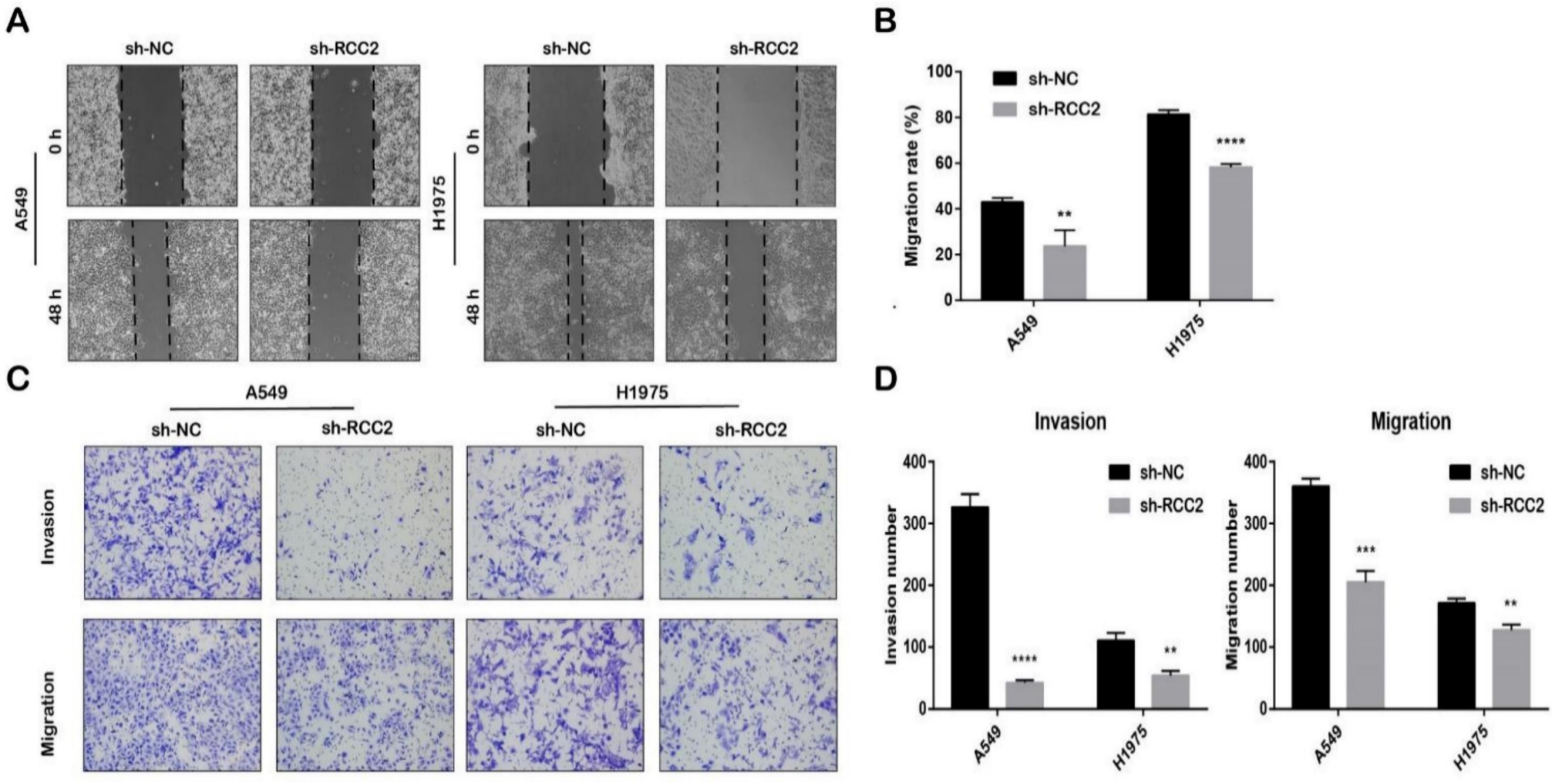
Down-regulation of RCC2 suppressed the migration and invasion abilities of A549 and H1975. (A-B) Migration distance of sh-RCC2 cells were significantly shorter than sh-NC cells. (** means p < 0.01, **** means p < 0.0001) (C-D) Transwell assay was used to evaluate the migration and invasion ability difference between sh-NC cells and sh-RCC2 cells. (** means p < 0.01, *** means p < 0.001, **** means p < 0.0001).

As shown in **Figure 8A**, downregulation of RCC2 expression remarkably shorten the migration distance of A549 (P<0.01) and H1975(P<0.0001). We also found that the relative number of migrated cells were significantly reduced after knocking down RCC2 expression in transwell assay **(Figure 8B)**. This result was consistent with scratch experiment **(Figure 8C-D)**. To sum up, downregulation of RCC2 could supress the migration and invasion abilities of LUAD cell.

## Discussion

Accumulated studies indicated that RCC1 and RCC2 played an indispensable role in progress of cell cycle regulation 34. RCC1 was proved to be a regulator in monitoring the process of DNA synthesis and through a Ran-dependent manner to link its completion with the occurrence of mitosis 35, 36. During nuclear membrane disintegration, RCC1 through keeping the gradient of RanGTP concentration around chromosomes to ensure normal mitosis 37. RCC1 deficiency would lead to the abnormal nuclear morphology in the end-stage of G1. RCC2 is one component of chromosomal passenger complex (CPC) that could regulate chromosomal alignment, spindle assembly, and cell cleavage during mitosis 38. Different from RCC1 only implicated in G1, downregulation of RCC2 not only lead to abnormal cell cycle progression of G1/S but also suppresses G2/M 39. Both RCC1 and RCC2 may form a complex through recognizing the DNA damage or regulating the DNA replication to prevent cell concertation. RCC1 and RCC2 have been confirmed to play key roles in many cancers 40, 41. With the help of multiple databases, we had revealed that RCC1 and RCC2 mRNA expression level and protein level were dramatically up-regulated in LUAD tissue compared with normal tissue. Our result also suggested that patients with the higher RCC1 and RCC2 expression levels would have shorter OS. RCC1 overexpression was also associated with shorter FPS and PPS. Furthermore, RCC2 expression level was validated by RT-PCR and IHC. Unfortunately, the samples size for these experiments were small, we will collect more samples to verify the current results in the next step of study.

In tumorigenesis of LUAD, DNA damage, abnormal replication and transcription mechanisms of DNA are important reasons of cancer initiation and chemoresistance 42. Spontaneous or drug-treated DNA damage result in impairment of nuclear cytoplasmic transport (NCT). The regulation of the Ran system enlarges the effect, which decelerates or pauses the process of cell cycle. 34. Once the mutation or aberrant activation of key molecules in the DNA repair signaling pathway could lead to the chemoresistance and tumor progression 43-45. Among these signaling pathway, TP53 is the most frequently altered gene in human malignant tumors 46. Once cells suffering from various stress, p53 would be activated and combined with chromatin to mediate the transcription of its downstream genes^47^, ^48^. Upon activation, p53 can mediate diverse cellular process through the downstream targets' functions, such as apoptosis, cell cycle arrest, autophagy, and DNA repair 49, 50. RCC1 superfamily proteins, especially RCC1 and RCC2, can monitor the DNA synthesis and mitosis through a Ran-dependent manner. Therefore, we hypothesized that there is some correlation between RCC1 superfamily proteins and TP53 signaling pathway. With the help of ULCAN database, we demonstrated that LUAD patients with TP53 mutation have higher RCC1 and RCC2 expression level compare with patient with TP53 non-mutation. Moreover, GSEA analysis suggested that p53 signaling pathway was upregulated in the LUAD patients with RCC2 overexpressed. In addition, RCC2 upregulation showed significantly correlated with DNA replication, Nucleotide excision repair and cell cycle. Taken together, RCC1 and RCC2 may participate in cell cycle regulation through TP53 signaling pathway and thus regulate the proliferation activities of LUAD cells. Unfortunately, we did not prove this hypothesis due to time limitation and experimental constraints. In the following experiment, we would like to identify the signaling protein RCC2 interacts with and explore their functional mechanism in cell proliferation.

Previous studies have explored the mechanism of RCC1 and RCC2 in other kinds of cancers, which is mainly ascribed to its regulation of numerous signaling pathways. In this research, we detected the related genes that were possibly associated with RCC1 and RCC2 functions, following by constructing the interaction network. Riahi et al. reported that RCC1 mutation had the carcinogenic potential in breast cancer 41. Another research indicated that c-Jun directly modulate RCC1 expression level in papillomavirus-related cervical cancer 51. In addition, the downregulation of KPNB1 in advanced prostate cancer could effectively decrease the expression phosphorylation of RCC1, which lead to cycle arrest 52. In colorectal cancer, downregulation of RCC2 leads to microsatellite instable tumor arrest at the G2-M phase and amplify cell apoptosis 53. Gong et al. demonstrated that RCC2 through RalA signaling pathway to promote ovarian cancer cell proliferation, migration and inhibit apoptosis and cisplatin-resistance 54. Some studies indicated that RCC2 was participated in superoxide-induced cell death in lung and ovarian cancers 55, 56. However, the biological functions and underline mechanisms of RCC1 and RCC2 in LUAD remain to be elucidated. We carried out the functional enrichment analysis to explore the potential biological function and underline mechanism of RCC1 and RCC2 in LUAD. Consisting with previous studies, our result indicated that RCC1, RCC2 and their correlative genes played an important role in the process of nucleocytoplasmic transport, positive regulation of GTPase activity and regulation of cell cycle process. Although we used multiple online databases, it still hard to assure the accordance among different databases resulting from the population differences. In the subsequent work, we will conduct relevant rescue experiments at the cellular and animal levels to confirm the function of RCC1 and RCC2 and their correlated genes in the above biological activities.

In the last decades, numerous studies indicated that tumor-infiltrating immune cells (TICs) had great impact on predicting clinical outcome and developing immunotherapy 57. Immunological parameters were demonstrated to have better predictive value on clinical outcomes than TNM staging in several cancer 58-64. Although development of immune checkpoint inhibitors had greatly prolonged patient survival, the objective response rate to checkpoint blockade still low. 65. In order to explore whether RCC1 and RCC2 participate in the modulation of TICs activity, we examined the RCC1 and RCC2 expression distribution in normal lung tissue and immune cells through the single cell RNA analysis in HPA database. We demonstrated that RCC1 expression in normal lung tissue were enrich in alveolar cells and endothelial cells. RCC2 expression distribution were mainly in macrophages, granulocytes and T cells. Macrophages showed the highest expression level among all cells. To date, few studies have suggested the prognostic value of TICs in NSCLC. AI-Shibli et al. assessed the prognostic effect of epithelial and stromal lymphocyte infiltration in NSCLC and discovered a high number of stromal CD8+ and CD4+ cells were independent positive prognostic factors for disease-specific survival 66. Branislava Stankovic et al. reported that T cells was the dominated immune cell in the composition of NSCLC, of which CD4+T cells represented the dominant abundant population of T cells (25.9%) followed by CD8+T cells (21.7%) 67. According to the above result, we hypothesized that RCC2 may adjust tumorigenesis and progression by regulating the infiltration of immune cells. Through the TIMER database, RCC2 expression shown positive relevance with macrophage, neutrophil, T cell CD8+ and T cell CD4+ infiltration, although the correlation index is not high. Furthermore, we also observed that RCC2 mutation and the sCNA status can decrease the macrophage, neutrophil and T cell CD4+ infiltration distribution. Our result indicated that RCC2 might participate in tumor immune reaction through media immune cell infiltration. Nevertheless, these findings need to be verified in future investigations.

In addition, we assessed the association between RCC2 expression level and LUAD cell migration and invasion ability through knocking down the RCC2 mRNA level in A549 and H1975 cell. The result of the transwell assay and wound-healing assay demonstrated that downregulation of RCC2 could significantly suppress the LUAD cell migration and invasion. Due to time limitations, other biological function experiments are still unfinished. In the next step, more rescue experiments at the cellular and animal levels will be performed to evaluate the function of RCC2 in other biological functions and explore the underlying mechanisms.

## Conclusions

In summary, our results indicate that RCC1 and RCC2 expression level has a significant association with patients' prognosis, TP53 mutation and cancer immune microenvironment. According to the cell functional experiment results, RCC2 was considered as an impact factor in LUAD cell migration and invasion. It is indicated that RCC1 and RCC2 might mediate tumor progression and RCC2 may play an important role in regulation of immunotherapeutic effects on LUAD. Therefore, RCC1 and RCC2, serving important roles in tumor progression, have the potential to be prognostic biomarkers for LUAD.

## Supplementary Material

Supplementary figure.
