# Supplementary material for: Comprehensive analysis of prognostic value and immune infiltration of Regulator of Chromosome Condensation 2 in lung adenocarcinoma
Source: J Cancer. 2024 Feb 11;15(7):1901–15. doi: 10.7150/jca.91367 (PMC10905397; doi:10.7150/jca.91367)
Supplement: Supplementary file 1 — Supplementary figure. [file jcav15p1901s1.pdf]

Comprehensive analysis of prognostic value and immune infiltration of Regulator of Chromosome Condensation 2 in lung adenocarcinoma

Hai Lin[1,2,3+], Guofu Lin[1,2,3+], Lanlan Lin[1,2,3+], Jiansheng Yang[4] , Dongyong Yang[1,2] , Qinhui Lin[1,2] , Yuan Xu[1,2*] , Yiming Zeng[1,2*]

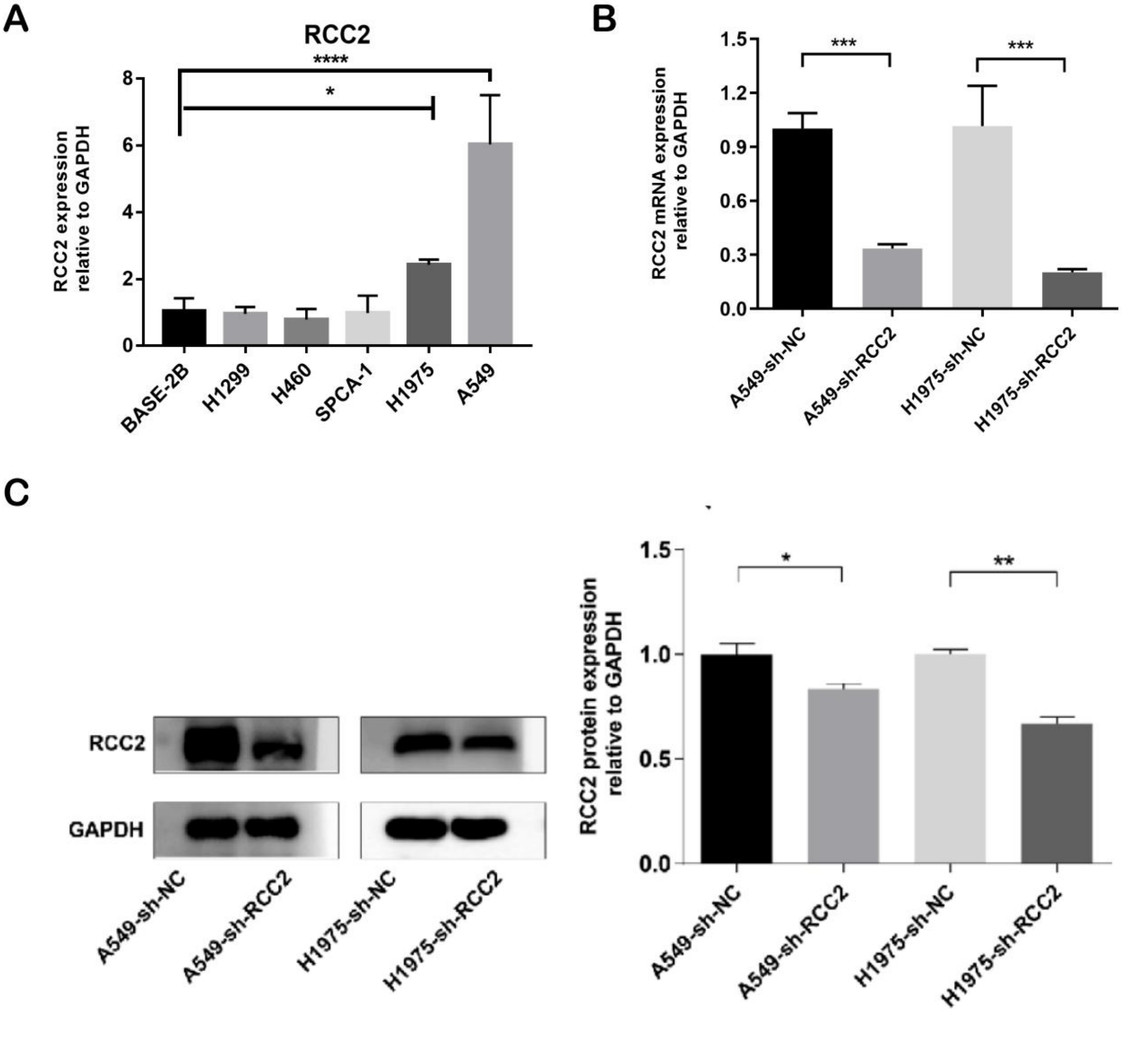

Figure S1. Construction of RCC2 knockdown stable transplanter cell line. (A) Detection of RCC2 mRNA expression level between normal airway epithelial cell (BASE-2B) and LUAD cell (H1299, H460, SPCA-1, A549 and H1975). (* means p＜0.05, **** means p＜0.0001) (B-C) Verifying

RCC2 expression level by qRT-PCR and Western blot between sh-NC cells and sh-RCC2 cells. (* means $p<0.05$, ** means $p<0.01$,*** means $p<0.001$)
